# FengLiao affects gut microbiota and the expression levels of Na+/H+ exchangers, aquaporins and acute phase proteins in mice with castor oil-induced diarrhea

**DOI:** 10.1371/journal.pone.0236511

**Published:** 2020-07-28

**Authors:** Wenlu Chen, Xinyu Peng, Jingxian Yu, Xuanxuan Chen, Minggui Yuan, Rong Xiang, Limei He, Danni Yu, Huahua Kang, Yufang Pan, Zhihong Xu

**Affiliations:** 1 Institute of Animal Health, Guangdong Academy of Agricultural Sciences, Guangzhou, China; 2 Guangdong Pharmaceutical University, Guangzhou, China; 3 Key Laboratory of Live stock Disease Prevention of Guangdong Province, Guangzhou, China; 4 Scientific Observing and Experimental Station of veterinary drugs and diagnostic Techniques of Guangdong Province, Ministry of Agriculture, Guangzhou, China; 5 Chinese Traditional Medicine Engineering Technology Research Center of Guangdong Province, Guangzhou, China; 6 South China Agricultural University, Guangzhou, China; University of Illinois at Chicago, UNITED STATES

## Abstract

The severe side effects of chemosynthetic anti-diarrhea drugs have created an interest in low-toxic alternative plant-derived compounds. FengLiao consists of *Polygonum hydropiper* Linn. and *Daphniphyllum calycinum* Bench., and is widely used in China to treat diarrhea due to low levels of toxicity. In this study, the effects of FengLiao were analyzed in a castor oil-induced diarrhea model, using the anti-diarrhea drug, loperamide, as the positive control. The effects were evaluated using stool characteristics and the expression levels of various diarrhea-related factors in the jejunum and liver, as well as changes in the microbiota of the jejunum. The symptoms of diarrhea and stool consistency were improved through FengLiao and loperamide treatment. Furthermore, FengLiao down-regulated alpha 1-acid glycoprotein (AGP) and C-reactive protein (CRP) levels, and up-regulated transferrin (TRF) mRNA levels in the liver, and down-regulated Aquaporin 3 (AQP3) and Na+/H+ exchanger isoform 8 (NHE8) expression in the epithelial cells of the jejunum. It also increased the relative abundance of *Bifidobacterium*, *Aerococcus*, *Corynebacterium*_1 and *Pseudomonas*, and lowered the Firmicutes/Bacteroidetes (F/B) ratio, which maintained the balance between immunity and intestinal health. Taken together, FengLiao alleviated castor oil-induced diarrhea by altering gut microbiota, and levels of jejunum epithelial transport proteins and acute phase proteins.

## Introduction

Traditional herbal medicines are still widely used in Asia for the treatment of diarrhea, especially in China, Japan and Korea. For example, the FengLiao formulation, which consists of *Polygonum hydropiper* Linn. and *Daphniphyllum calycinum* Bench., is used as an analgesic, antispasmodic and anti-immune agent to treat diarrhea and gastric ulcers [1–6]. However, the molecular mechanisms underlying the anti-diarrheal effects of FengLiao have still not been elucidated.

Diarrhea is a common clinical sign of gastrointestinal disease, and is characterized by frequent watery stools and abdominal pain. It is usually the results of infection, food poisoning or chemotherapy. More than one million cases of diarrhea are diagnosed per year in both developed and developing countries, and it is a leading cause of death among children younger than five years of age [7, 8]. Anti-diarrheal drugs include drugs that balance electrolytes, antimotility drugs and antibiotics. Herbal medicines have attracted increasing levels of attention due to low levels of toxicity and fewer side effects [9–12]. Diarrhea can be induced in animal models through many methods, including bacterial infection [13], calcitonin gene-related peptide (CGRP) [14], lipopolysaccharide (LPS) and castor oil [15]. The castor oil-induced diarrhea model has been routinely used due to reproducibility, stability and a lack of the risk of infection. The main active constituent in castor oil is the C-18 hydroxy fatty acid ricinoleic acid, which can induce diarrhea by impairing circular muscles and the surface of epithelial cells, and increasing the secretion of fluids and electrolytes into the gastric lumen [16].

Studies have shown that aquaporins (AQPs) [17–19] and Na+/H+ exchangers (NHEs) [20–24] are critical for the absorptive and secretory function of the gastric epithelia. In addition, serum levels of acute phase proteins (APPs) [25, 26] are correlated with the severity of diarrhea and intestinal inflammation. Dysregulation of AQPs is an auxiliary pathological factor in certain gastrointestinal diseases. For instance, AQP3 is up-regulated in some animal models of diarrhea [18], whereas AQP4 level is down-regulated in inflammatory bowel disease patients [19]. Na+/H+ exchangers play key roles in Na+ absorption in the gastrointestinal tract, and are often impaired in acute and chronic diseases. NHE2 is relatively widely expressed [20], and its deficiency altered acid secretion in the intestinal mucous layer and impaired the recovery of the intestinal barrier [23]. NHE3-knockout mice exhibited a severe sodium absorptive defect in the intestine, along with mild diarrhea, while NHE8 could compensate for the loss of NHE2 and NHE3 to exert a protective effect on gut mucosa [21, 24]. APPs, such as alpha 1-acid glycoprotein (AGP), transferrin (TRF), albumin (ALB) and C-reactive protein (CRP), are produced by hepatocytes as part of the innate acute phase response [25] to trauma, infection, stress, neoplasia and inflammation [26], and their expressions are elevated in diarrhea.

Growing evidence has shown that the gut microbiota, a diverse enriched microbial ecosystem that contains nearly 100 trillion bacteria [27, 28], participates in biological activities and affects physiological functions of the gastrointestinal tract. The gut microbiota is also known as “the forgotten organ” [29] and it can have a large impact on the treatment of infectious diseases through the production of antibiotics, regulation of immune and responses of pro-inflammatory, which prevent the invasion of pathogens by functioning as a barrier [30–32], by influencing general health through the bio-synthesis of vitamins and amino acids, and also by modulating susceptibility to infectious diseases [33, 34]. Therefore, investigation of the effects of FengLiao on gut microbiota is not only valuable but also essential.

In this study, the effects of FengLiao were analyzed using the expression levels of multiple diarrhea-related factors in gut microbiota of a castor oil-induced diarrhea mouse model.

## Materials and methods

### Preparation of FengLiao and standards

The leaves and branches of *Daphniphyllum calycinum* Bench. and the grass of *Polygonum hydropiper* Linn. were purchased from Dashenlin Pharmacy (Guangzhou, Guangdong, China). The identity of the plant materials were verified by Professor Xuan-xuan Cheng, a Pharmacologist specializing in Chinese Traditional Medicine. FengLiao was prepared as previously described [6]. In brief, 40 g of *Daphniphyllum calycinum* Bench. and 20 g of *Polygonum hydropiper* Linn. were refluxed and extracted twice in 10 volumes of 65% ethanol (v/w) for 60 min each time, and then the extracts were filtered. Both extracts were combined and concentrated under vacuum until an alcohol smell was not detected. The solution was diluted with distilled water to the appropriate concentrations and filtered through a 0.22 μm membrane filter. To prepare the standards, 10 mg each of rutin, hyperoside, quercitrin, quercetin, isorhamnetin and kaempferol were dissolved separately in methanol, and dispensed into 10 ml volumetric bottles. Thereafter, appropriate volumes of the compounds were aliquoted into five 5 ml volumetric bottles, and each was made to final concentrations of 0.1 μg/ml, 1 μg/ml, 10 μg/ml, 50 μg/ml and 100 μg/ml.

### Chromatographic analysis

The above preparations were analyzed using a Waters series high performance liquid chromatography (HPLC) system equipped with a Waters 2489 UV detector, 515 HPLC Pump and 717 plus Autosampler. Chromatographic separation was performed on a Waters Symmetry shield RP18 column (4.6 mm x 250 mm, 5 μm) at 30°C. The flow rate was set at 1 ml/min and the injection volume was 10 μl. The solvents, acetonitrile (A) and 0.4% aqueous formic acid (B), were applied at the following gradient: 0–15 min, 20% A-35% A; 15–20 min, 35% A- 55% A; 20–25 min, 55% A-60% A; 25 min, 60% A-70% A; 30–35 min, 70% A-20% A.

### Establishment of a diarrhea model and treatment regimen

Three to four-week-old wild-type KM mice weighing 20–22 g were obtained from Jinan Pengyue Experimental Animal Breeding Co. Ltd. (Shandong, China), and housed in an SPF (specific pathogen free) room at constant temperature (23±1.5°C) and humidity (50±15%) under 12 h light/dark cycles. The food and padding were sterilized with ^60^Co provided by Beijing Huafukang Biotechnology Co. Ltd. All experiments were approved by the Animal Care Committee of the Institute of Guangdong Pharmaceutical University. The dose of FengLiao to be used was determined through our previous study [6], and the dosage of loperamide used was based on a previously published report [35]. The dosage of castor oil that induced diarrhea in 100% of the mice without causing death was determined in a pilot experiment. The mice were adaptively fed for 7 days and then randomly divided into the following ten groups (10 mice in each, 5 males and 5 females): (1) untreated control (Control), (2) untreated castor oil-induced model (Model), (3) loperamide-treated model (CastorOil_LP), (4) loperamide control (LP), (5) high dose FengLiao (H), (6) medium dose FengLiao (M), (7) low dose FengLiao (L), (8) high dose of FengLiao-treated model (CastorOil_H), (9) medium dose of FengLiao-treated model (CastorOil_M), and (10) low dose of FengLiao-treated model (CastorOil_L). Mice in all FengLiao treatment groups (group 5, 6, 7, 8, 9 and 10) were gavaged with 0.4 ml of 1 g/ml (H), 0.5 g/ml (M) and 0.25 g/ml (L) FengLiao extract. Mice in the untreated control and model group (group 1 and 2) were gavaged with 0.4 ml of saline, and mice in the LP treatment groups (group 3, 4) were administered 5 mg/kg LP. The mice were gavaged with the appropriate solutions once a day for three days, and food was withheld on the second night after administration. Diarrhea was induced in all the model groups (group 2, 3, 8, 9 and 10) on the third day through the gavage of 0.5 ml castor oil, 30 min after the respective drug was administered.

### Sample collection

**Fig 1:**
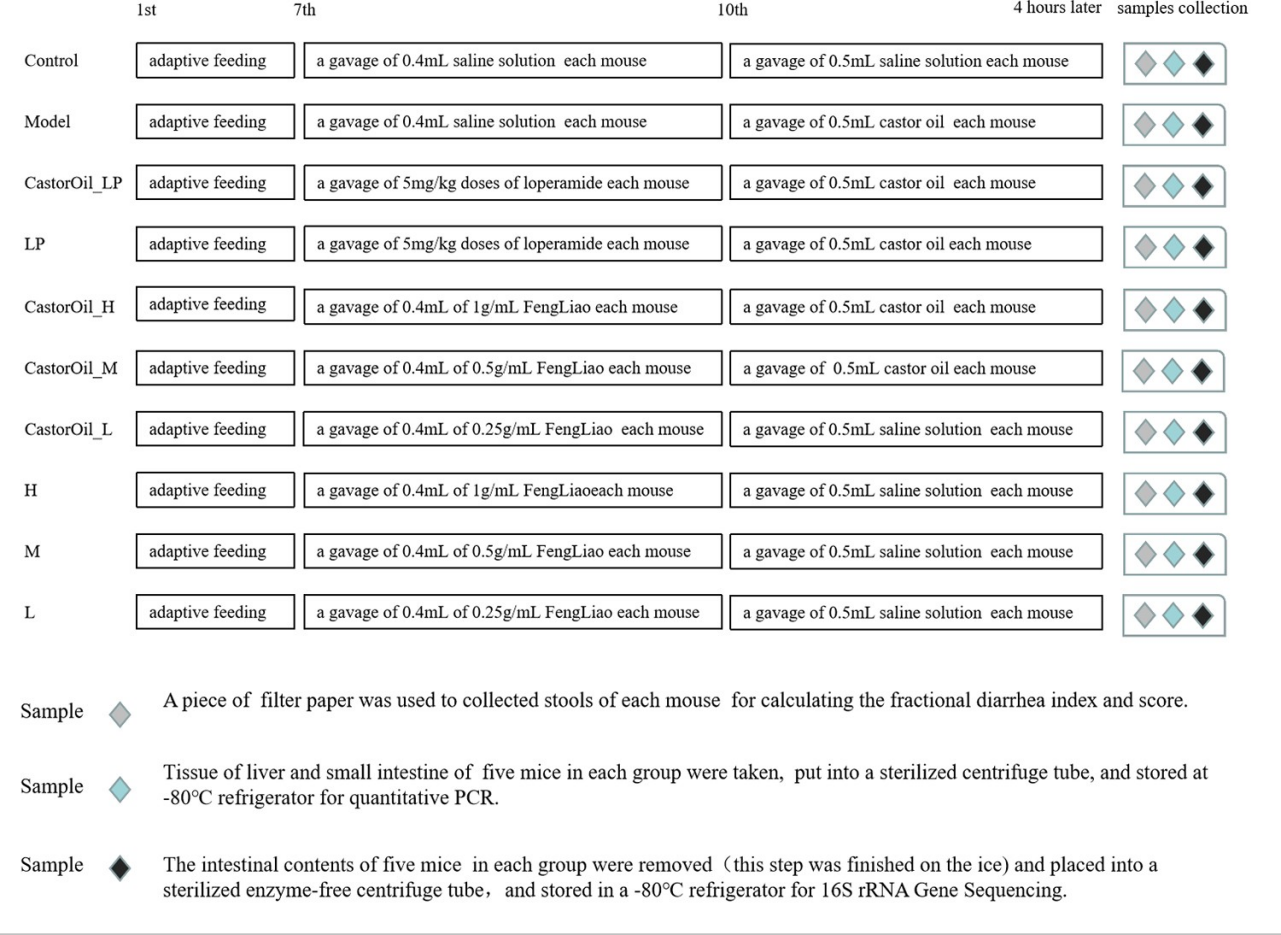
Experimental design and sample collection.

Following the induction of diarrhea, the mice were housed separately and the stool of each mouse was collected on a piece of filter paper. Four hours later, the mice were euthanized through cervical dislocation. Five mice were randomly selected from each group and intestinal contents were removed and instantly frozen on dry ice, and stored at -80°C. Liver and jejunum samples were obtained from the remaining five mice in each group and frozen at -80°C. The experimental design and sample collection are outlined in Fig 1.

### Diarrhea scoring

The stools collected were individually scored on the level of diarrhea based on their consistency as follows: 0: normal stool, 1: soft, 2: soft and slightly wet, 3: wet and unformed stool with moderate perianal coating, and 4: watery stool with severe perianal coating. The diluted/watery stools were graded as follows: 1: diameter less than 1 cm, 2: 1–1.9 cm, 3: 2–3 cm, and 4: > 3 cm. The stool diameter was measured for the uniformly round shape, and for samples with irregular shape, the longest and shortest diameters were measured and the average was calculated. The dilute level of the stool of each mouse was calculated as the sum of the dilute stool grades divided by the number of dilute stools, while the dilute stool rate of each mouse was calculated as the number of dilute stools divided by the total number of stools, which was then multiplied by 100. Finally, the diarrhea index was calculated as the dilute stool rate multiplied by the dilute stool level.

### RNA isolation and real time-quantitative PCR(QPCR) of the diarrheal-related genes

Total RNA was extracted from jejunum and liver tissues using TRIzol reagent and quantified using a NanoDrop® ND-2000 UV spectrophotometer (NanoDrop Technologies, United States). RNA was dissolved in DEPC-treated water and stored at −80°C. Reverse transcription was performed using a PrimeScriptTM RT reagent Kit with gDNA Eraser (TAKARA Bio, Japan), according to the manufacturer’s protocol. Quantitative PCR was performed in triplicate using SYBR Premix Ex Taq (TAKARA Bio, Japan). The target gene expression levels were normalized to that of β-actin. The relative expression level of each target gene was calculated using the 2^−ΔΔCt^ method, where -ΔΔCT = -(ΔCT_experimental group_ -ΔCT_control group_) and ΔCT = CT_sample_—CT_β-actin_. The primers specific for NHE8, NHE3, NHE2, AQP3, AQP4, AGP, CRP, TRF, ALB and β-actin are listed in Table 1.

**Table 1:** The primer sequences.

Gene	Primer Forward(5'-3')	Primer Reverse(5'-3')	Product Length(bp)
AQP3	GAGATGCTTCACATCCGCTACCG	CCAGCCACCAAGATGCCAAGG	188
AQP4	GGAGCTACATGGAGGTGGAGGAC	GTCTCTTCTGCCTTCAGTGCTGTC	179
NHE2	TTACCTAAGAACACAAAGCTTCCAG	GAGCACAGTGGTTCCAACATC	108
NHE3	CTTCGCCTTCCTGCTGTCCTTG	GCCGACTGCTCTGAGATGTTGG	195
NHE8	CTTCTTCACACGGCGGCTGAC	CCTGACGGACCTCCTCATACCAC	95
AGP	CCAGAAGGCTGTCACACACG	GCTTCTTCTCCTGCTGACCG	92
CRP	CAGAGATTCCTGAGGCTCCAACA	AGTCACCGCCATACGAGTCCTG	177
ALB	CTACAGCGGAGCAACTGAAGACTG	GGTGTCCTTGTCAGCAGCCTTG	83
TRF	GCTGCTCCTCCACTCAACCATTC	CCTCATACTGATCCACTGGCTTGC	186
β-actin	GTGCTATGTTGCTCTAGACTTCG	ATGCCACAGGATTCCATACC	174

### 16S rRNA gene sequencing

Genomic DNA was extracted from mice stools using a DNA extraction kit, according to the manufacturer’s instructions. The 16S V3 and V4 regions were amplified using the primers, 338F (5’-ACTCCTACGGGAGGCAGCAG-3’) and 806R (5’-GGACTACHVGGGTWTCTAAT-3’). The PCR conditions applied were as follows: one pre-denaturation cycle at 95°C for 3 min, 27 cycles of denaturation at 95°C for 30 s, annealing at 55°C for 30 s and elongation at 72°C for 30 s and a post-elongation cycle at 72°C for 10 min. The PCR amplicons were separated on 2% agarose gels and then extracted using the AxyPrep DNA Gel Extraction Kit (Axygen Biosciences, United States), according to the manufacturer’s instructions. After purification using QuantiFluor™-ST (Promega, United States) to remove primer dimers, the resulting amplicons were pooled in equimolar concentrations and paired-end sequenced (2 x 300) on an Illumina MiSeq platform (Illumina, United States) according to the standard protocols by Majorbio Bio-Pharm Technology Co. Ltd. (Shanghai, China). The raw fastq files were demultiplexed, and high quality reads were filtered using Trimmomatic and merged using FLASH based on the following criteria: (1) truncated at any site with an average quality score < 20 over a 50 bp sliding window, (2) mismatch of only two nucleotides between primers and sequences, allowing for the removal of reads containing ambiguous bases, and (3) merging of sequences with > 10 bp overlap. The tags were clustered into operational taxonomic units (OTUs) using USEARCH (v7.0.1090) software, and representative sequences were taxonomically classified using Ribosomal Database Project Classifier v2.2 trained on the Greengenes database.

### Statistical analysis

The relative mRNA expression, relative abundance of intestinal flora, alpha diversity of the Shannon index and abundance-based coverage (ACE) were tested using single-factor analysis of molecular variance ANOVA. Genus and species level abundance were compared using the Kruskal-Wallis test with Benjamini-Hochberg p-value correction. P values less than 0.05 were considered to be statistically significant.

## Results

### Characterization of FengLiao components

Based on the linear relationship between peak area (Y) and concentration (X) (Table 2), rutin, hyperoside, quercitrin, quercetin, isorhamnetin and kaempferol were determined to be the six main components of FengLiao and were found to constitute 3.49%, 1.81%, 0.45%, 1.73%, 0.15% and 1.69% of the preparation (Table 3).

**Table 2:** A linear relationship between peak area (Y) and concentration (X).

Components	Regression Equation	Linearity/(μg/mL)	R^2^
Rutin	y = 20785x-1885.8	0.1~100	0.9998
Hyperoside	y = 19860x+5368.9		0.9997
Quercitrin	y = 16884x+6745		0.9998
Quercetin	y = 32354x+1850		0.9998
Isorhamnetin	y = 38458x-8908		0.9999
Kaempferol	y = 39924x-4444.3		0.9999

**Table 3:** The content of active components of FengLiao.

Component	Rutin	Hyperoside	Quercitrin	Quercetin	Isorhamnetin	Kaempferol
Content(mg/g)	0.97	0.51	0.13	0.48	0.04	0.47
	0.98	0.51	0.12	0.49	0.04	0.47
	0.97	0.51	0.13	0.48	0.04	0.47
	0.98	0.51	0.13	0.48	0.04	0.47
	0.98	0.50	0.13	0.48	0.04	0.47
	0.98	0.50	0.13	0.49	0.04	0.47
The percentage of the total material	3.49%	1.81%	0.45%	1.73%	0.15%	1.69%

Each kilogram dried FengLiao extraction can be dried into 27.94g total material.

### Establishment of a castor oil-induced diarrhea mouse model

**Fig 2:**
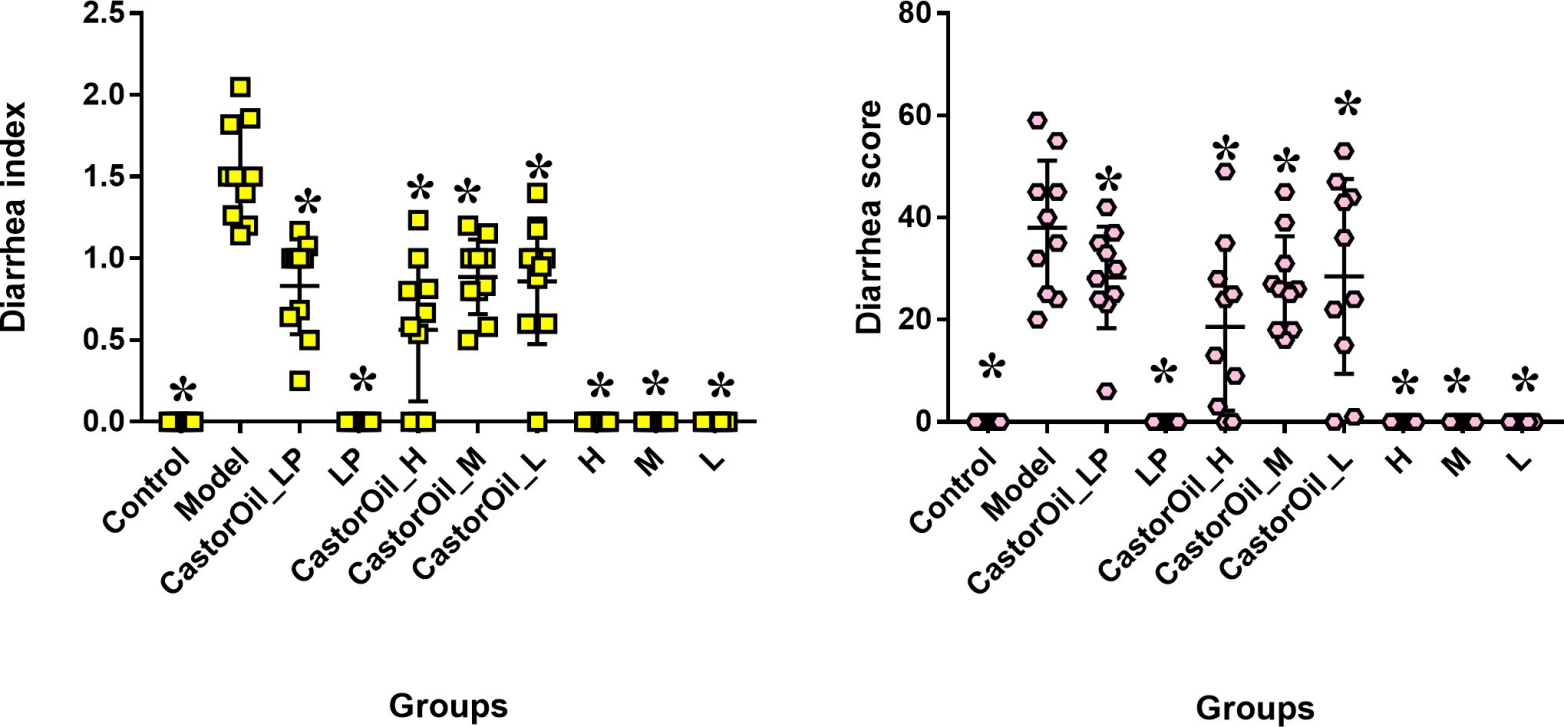
Estimates of diarrhea index and diarrhea score(n = 10). The effect of castor oil administration diarrhea score and index of mice. Castor oil increased the diarrhea score and index, while FengLiao decreased them. LP (loperamide), H (high dose of FengLiao), M (medium dose of FengLiao), L (low dose of FengLiao). 1 g/ml FengLiao (H), 0.5 g/ml FengLiao (M), 0.25g/ml FengLiao (L) and 5mg/kg loperamide were pretreated in CastorOil_H, H, CastorOil_M, M, CastorOil_L, L, CastorOil_LP, LP groups once a day for three days respectively. After 30 min at last times drug administration, 0.5ml castor oil was used to induce diarrhea in CastorOil_H, CastorOil_M, CastorOil_L and CastorOil_LP groups for 4 h and the stool characteristics were observed and calculated. Y axis shows diarrhea index or diarrhea score. X axis represents groups’ name. *p<0.05 versus model group, ANOVA Test.

The diarrhea score and diarrhea index were significantly higher in the untreated model group compared with the control group and the drug-treated model groups. This indicated that a diarrhea model had been successfully established, with both FengLiao and loperamide having produced an obvious therapeutic effect (Fig 2).

### FengLiao alters the expression pattern of diarrhea-related factors in the jejunum

**Fig 3:**
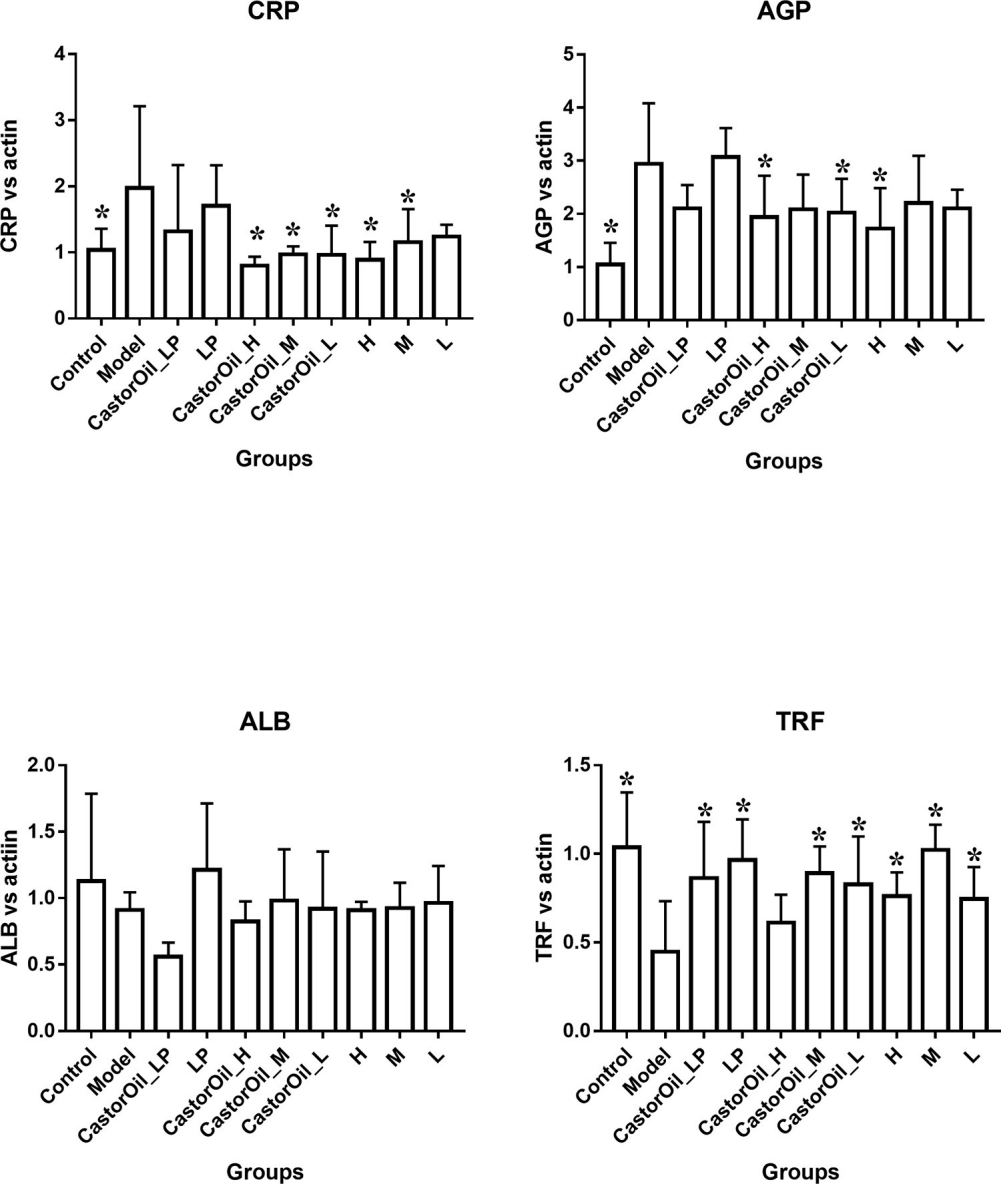
The expression of APPs mRNAs in the liver. Changes in acute phase proteins (APPs) mRNA expression in the liver of mice after castor oil administration. Castor oil increased the mRNA expression of CRP and AGP, but decreased that of TRF. And FengLiao restored them to normal levels. LP (loperamide), H (high dose of FengLiao), M (medium dose of FengLiao), L (low dose of FengLiao). 1 g/ml FengLiao (H), 0.5 g/ml FengLiao (M), 0.25g/ml FengLiao (L) and 5mg/kg loperamide were pretreated in CastorOil_H, H, CastorOil_M, M, CastorOil_L, L, CastorOil_LP, LP groups once a day for three days respectively. 30 min after the last drug administration, 0.5ml castor oil was used to induce diarrhea in CastorOil_H, CastorOil_M, CastorOil_L and CastorOil_LP groups for 4 h and the transcriptional level of expression for APPs was determined by qRT-PCR for CRP, AGP, ALB and TRF. Bar graphs show quantitative results normalized to β-actin mRNA levels. X axis represent groups’ name. Column represents the mean±SD of five mice. *p<0.05 versus model group, ANOVA Test.

**Fig 4:**
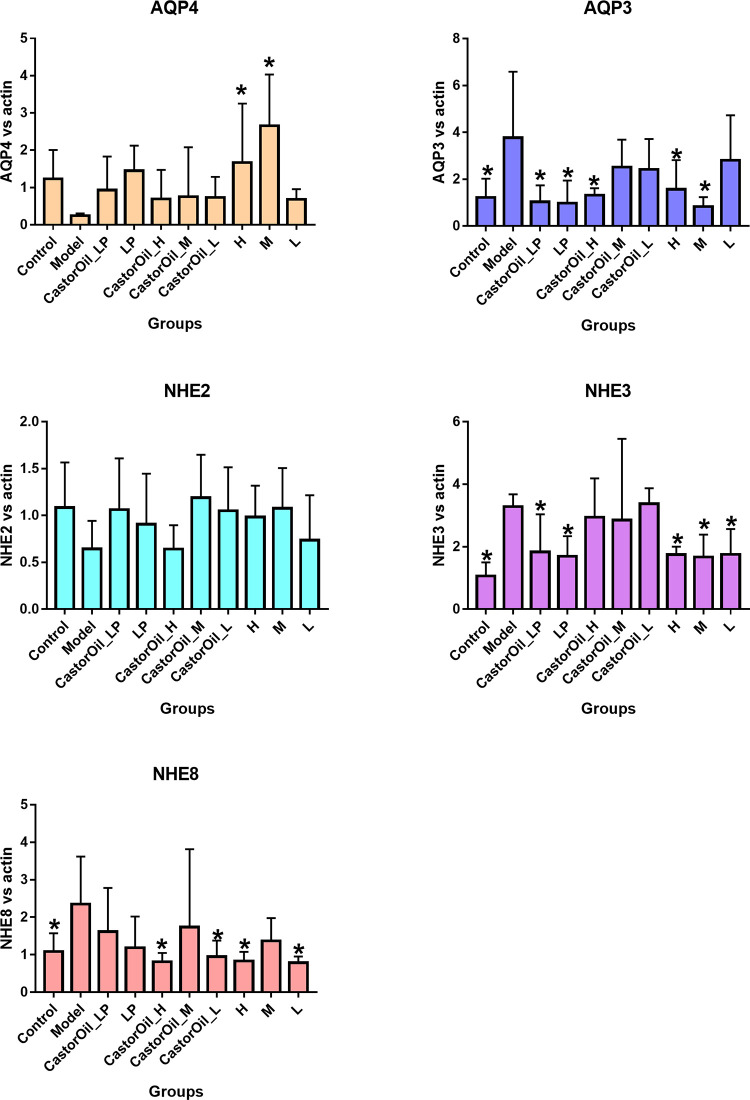
NHEs and AQPs mRNA levels in the small intestines. Changes in diarrheal associated genes expression in the jejunum of mice after castor oil administration. Castor oil increased the mRNA expression of AQP3, NHE3 and NHE8, while FengLiao decreased that of AQP3 and NHE8. LP (loperamide), H (high dose of FengLiao), M (medium dose of FengLiao), L (low dose of FengLiao). 1 g/ml FengLiao (H), 0.5 g/ml FengLiao (M), 0.25g/ml FengLiao (L) and 5mg/kg loperamide were pretreated in CastorOil_H, H, CastorOil_M, M, CastorOil_L, L, CastorOil_LP, LP groups once a day for three days respectively. 30 min after the last drug administration, 0.5ml castor oil was used to induce diarrhea in CastorOil_H, CastorOil_M, CastorOil_L and CastorOil_LP groups for 4 h and the transcriptional level of expression for diarrhea associated genes was determined by qRT-PCR for AQP3, AQP4, NHE2, NHE3, NHE8. Bar graphs show quantitative results normalized to β-actin mRNA levels. X axis represent groups’ name. Column represents the mean±SD of five mice. *p<0.05 versus model group, ANOVA Test.

The administration of castor oil significantly increased AGP and CRP mRNA levels in the liver, down-regulated TRF expression but had no effect on ALB expression. Pre-treatment with FengLiao significantly reversed the expression levels of these APPs. In addition, upregulation of AQP3, NHE3 and NHE8 were observed in the untreated model group, compared with the untreated control group. Downregulation of AQP3 and NHE8 expressions were observed in the FengLiao-treatment model groups, but no significant change was found in NHE3 expression. In contrast, loperamide exerted an ameliorative effect on all three markers. AQP4 and NHE2 expression levels were not significantly affected by castor oil treatment (Figs 3 and 4).

### Microbiota composition

**Fig 5:**
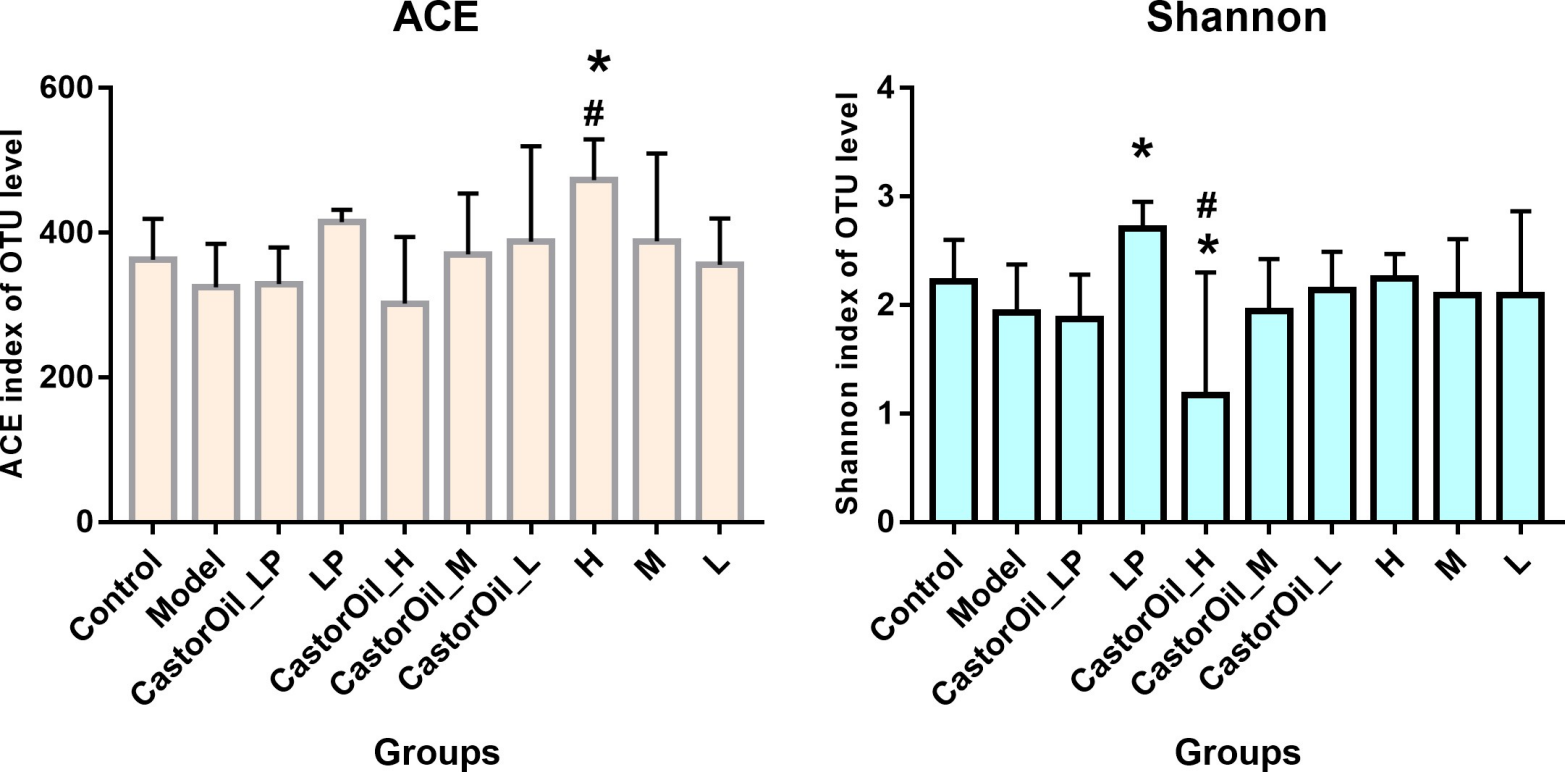
Evaluation of alpha diversity for the gut microbiota from intestine content. LP (loperamide), H (high dose of FengLiao), M (medium dose of FengLiao), L (low dose of FengLiao). 1 g/ml FengLiao (H), 0.5 g/ml FengLiao (M), 0.25g/ml FengLiao (L) and 5mg/kg loperamide were pretreated in CastorOil_H, H, CastorOil_M, M, CastorOil_L, L, CastorOil_LP, LP groups once a day for three days respectively. 30 min after the last drug administration, 0.5ml castor oil was used to induce diarrhea in CastorOil_H, CastorOil_M, CastorOil_L and CastorOil_LP groups for 4 h and the intestinal contents were sequenced for bacterial 16S rRNA. Bar graphs show ACE index and Shannon index of OTU level. X axis represent groups’ name. Column represents the mean±SD of five mice.versus model group: *p < 0.05, versus control group #p<0.05, ANOVA Test.

**Fig 6:**
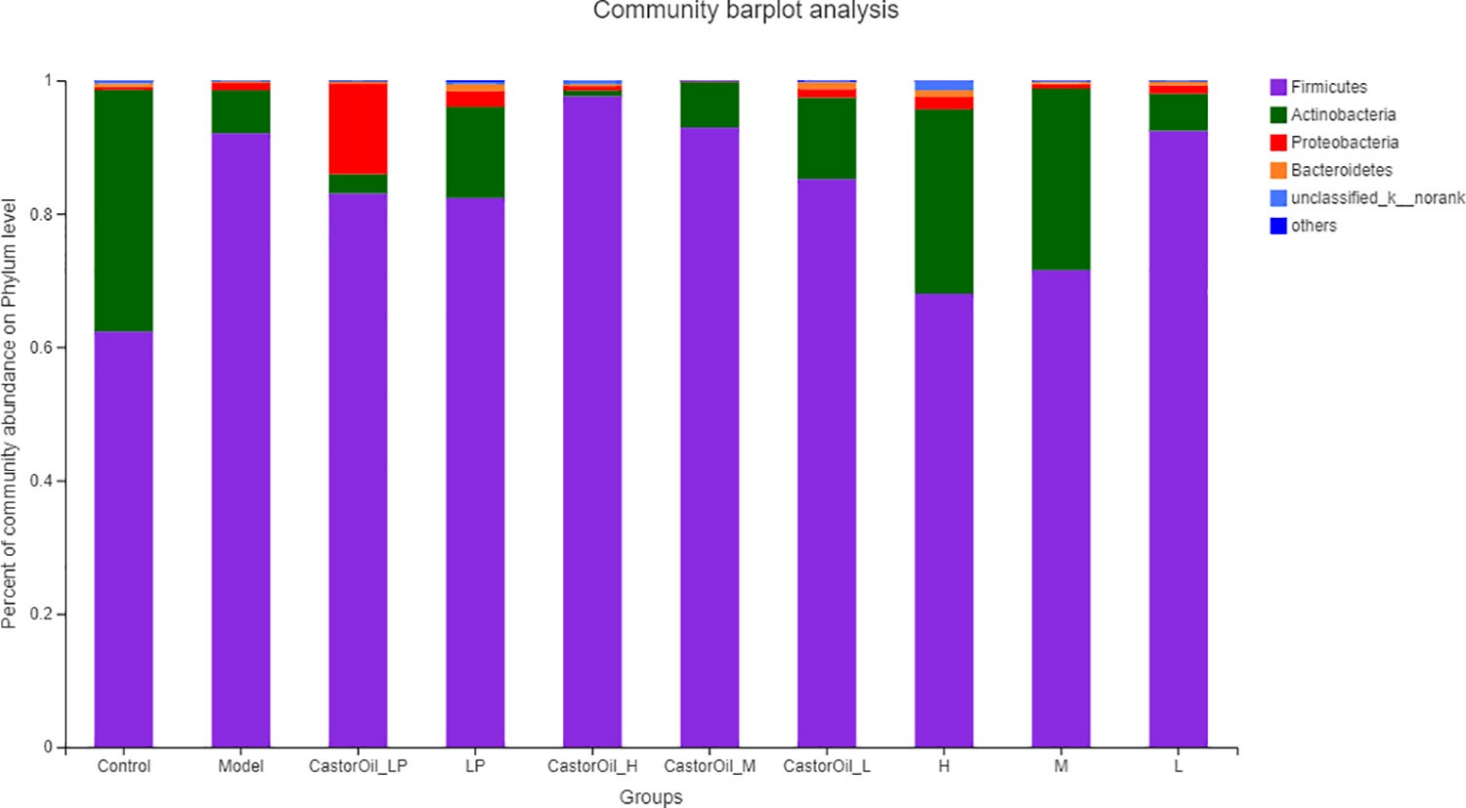
Bar plot analysis of phylum level communities in phylum level. LP (loperamide), H (high dose of FengLiao), M (medium dose of FengLiao), L (low dose of FengLiao). 1 g/ml FengLiao (H), 0.5 g/ml FengLiao (M), 0.25g/ml FengLiao (L) and 5mg/kg loperamide were pretreated in CastorOil_H, H, CastorOil_M, M, CastorOil_L, L, CastorOil_LP, LP groups once a day for three days respectively. 30 min after the last drug administration, 0.5ml castor oil was used to induce diarrhea in CastorOil_H, CastorOil_M, CastorOil_L and CastorOil_LP groups for 4 h and the intestinal contents were sequenced for bacterial 16S rRNA. Bar graph shows percent of community abundance on phylum level.X axis represent groups’ name, Column represents the mean of five mice.

**Fig 7:**
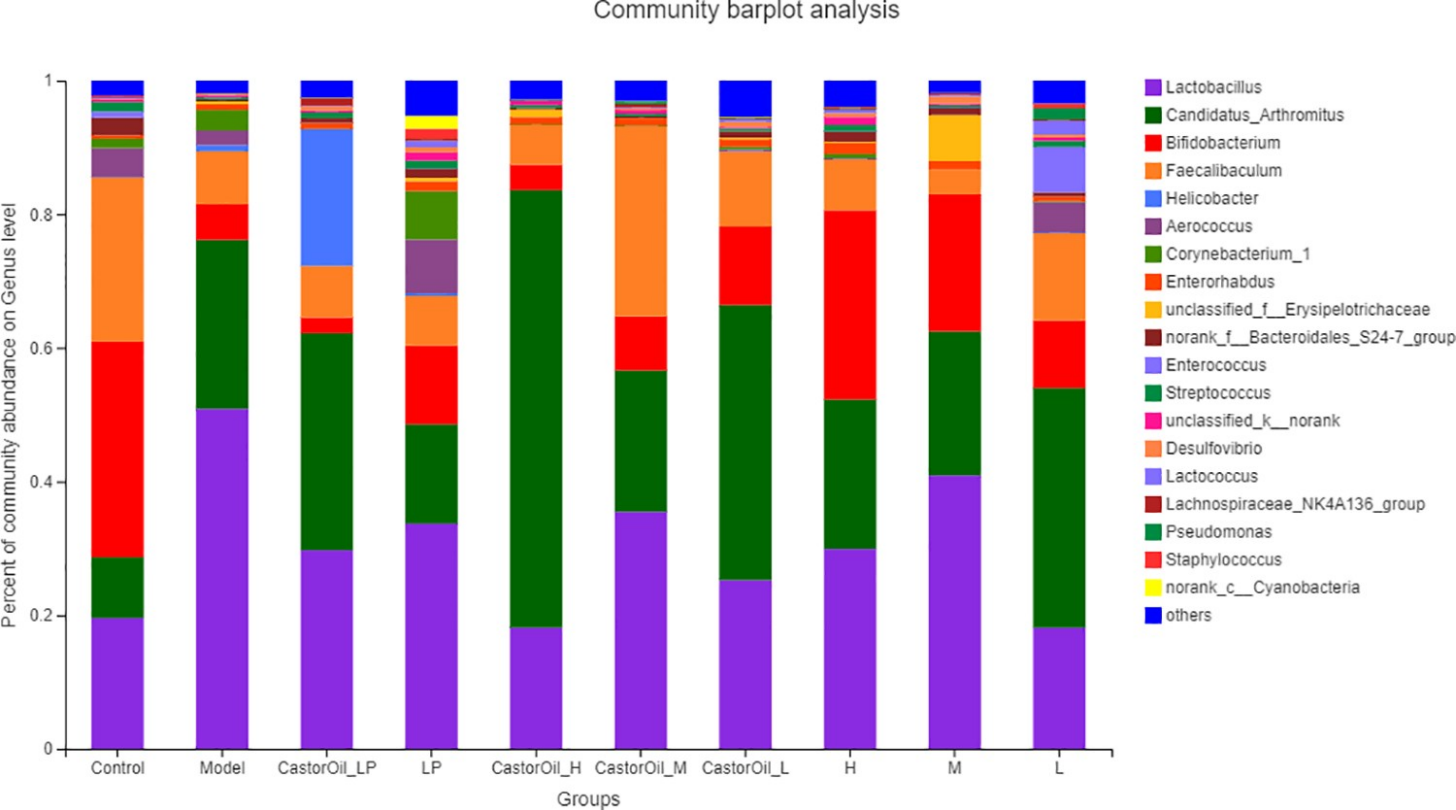
Bar plot analysis of genus level communities in genus level. LP (loperamide), H (high dose of FengLiao), M (medium dose of FengLiao), L (low dose of FengLiao). 1 g/ml FengLiao (H), 0.5 g/ml FengLiao (M), 0.25g/ml FengLiao (L) and 5mg/kg loperamide were pretreated in CastorOil_H, H, CastorOil_M, M, CastorOil_L, L, CastorOil_LP, LP groups once a day for three days respectively. 30 min after the last drug administration, 0.5ml castor oil was used to induce diarrhea in CastorOil_H, CastorOil_M, CastorOil_L and CastorOil_LP groups for 4 h and the intestinal contents were sequenced for bacterial 16S rRNA. Bar graph shows percent of community abundance on genus level. X axis represent groups’ name, Column represents the mean of five mice.

The alpha diversity of the intestinal microbiota in the different groups were assessed in terms of Shannon and ACE index (evenness and richness) (Fig 5). FengLiao significantly increased the ACE index, which is indicative of microbiota richness, in a dose-dependent manner, compared with that of control and untreated diarrheal mice. However, the Shannon index was significantly lower in the CastorOil_H group than in the untreated model group and the control group, indicating that FengLiao may have had decreased the evenness of the intestinal microbial community in castor oil-induced diarrheal mice. The predominant phyla in the gut were Cyanobacteria, unclassified_k_norank, Bacteroidetes, Proteobacteria and Actinobacteria, while Firmicutes was the dominant phyla (Fig 6). The Firmicutes/Bacteroidetes (F/B) ratios of both the untreated model group and the high-dose FengLiao-treated model group were higher than that of other groups (Table 4). Interestingly, high dose of FengLiao decreased the relative abundance of Actinobacteria in normal mice, while lower doses produced an opposite effect. In addition, FengLiao increased the relative abundance of Bacteroidetes. The predominant genera in the gut microbiota of all groups were *Lactobacillus*, *Candidatus_Arthromitus*, *Bifidobacterium*, *Faecalibaculum*, *Helicobacter*, *Aerococcus*, *Corynebacterium_1*, *Enterorhabdus*, *unclassified_f_Erysipelotrichaceae*, *norank_f_Bacteroidales_S24-7_group*, *Enterococcus*, *Streptococcus*, *unclassified_k_norank*, *Desulfovibrio*, *Lactococcus*, *Lachnospiraceae_NK4A136_group*, *Pseudomonas*, *Staphylococcus* and *norank_c_Cyanobacteria*. Both castor oil and treatment drugs altered the composition of the bacterial flora (Fig 7), while FengLiao treatment greatly increased the relative abundances of *Bifidobacterium*, *Aerococcus*, *Corynebacterium_1* and *Pseudomonas* (Table 4). All raw sequences produced were submitted to the Sequence Read Archive database at NCBI (Accession No. PRJNA604475).

**Table 4:** The relative abundance of mice gut microbiota in various groups.

No	Species name	Taxonomy	Control	Model	CastorOil_LP	LP	CastorOil_H	CastorOil_M	CastorOil_L	H	M	L
1	Firmicutes	phylum	61.840±7.847	87.170±14.940	75.800±26.270	72.590±8.581	92.560±7.516	88.840±11.790	80.790±8.051	64.240±19.730	74.840±12.280	82.840±12.650
2	Actinobacteria	phylum	34.500±9.403	10.760±14.060	3.348±2.220##	20.140±11.040###***	5.757±7.579##	8.904±9.821#	15.100±9.501	30.690±20.940***	22.510±10.840###***	10.520±12.880###***
3	Proteobacteria	phylum	0.296±0.266	1.304±1.135	19.650±26.610	2.160±1.526	0.649±0.670	1.303±2.189	1.974±1.681	1.828±1.126	1.208±1.369	4.222±7.675
4	Bacteroidetes	phylum	2.835±4.383	0.313±0.314	0.763±1.172	1.569±1.599*	0.310±0.204	0.264±0.310	1.341±1.247	1.796±1.776##***	1.064±1.453*	1.396±1.764
5	unclassified_k__norank	phylum	0.352±0.109	0.239±0.321	0.186±0.277	1.452±2.541#*	0.570±0.550	0.476±1.029	0.207±0.168	1.225±1.017	0.257±0.256	0.480±0.686
6	Cyanobacteria	phylum	0.006±0.002	0.0757±0.111	0.033±0.030	1.861±4.081	0.058±0.098	0.080±0.159	0.100±0.167	0.055±0.068	0.022±0.022	0.031±0.054
7	Lactobacillus	genus	19.810±12.200	48.710±30.390	33.150±22.470	34.610±9.164	17.840±24.460	35.570±10.410	26.980±10.390	29.730±17.400	42.650±24.470	17.890±7.415
8	Candidatus_Arthromitus	genus	8.480±10.170	25.140±27.860	31.400±24.010	14.250±16.590	66.06±42.790#	23.680±22.140	36.450±27.480	23.070±20.540	20.100±31.220	36.950±34.680
9	Bifidobacterium	genus	32.460±10.220	6.086±8.539	2.375±2.130	11.630±8.949	3.700±4.911	7.435±8.795	12.760±8.198	27.610±19.770**	20.740±10.410	9.582±12.260
10	Faecalibaculum	genus	25.250±13.670	8.624±8.427	7.381±5.985	8.176±7.700	5.902±8.460	27.330±16.890	13.190±11.630	7.699±7.832	3.675±1.709	12.010±12.780
11	Helicobacter	genus	0.021±0.025	0.887±0.913	18.770±25.480	0.391±0.655	0.011±0.013	0.007±0.015	0.076±0.140	0.003±0.003	0.008±0.017	0.103±0.207
12	Aerococcus	genus	3.908±8.036	2.566±5.265	0.0187±0.026	7.764±8.974	0.002±0.002	0.002±0.002	0.259±0.315	0.106±0.183##**	0.014±0.008	3.931±8.194
13	Corynebacterium_1	genus	1.284±1.778	3.471±4.742	0.0419±0.075	6.750±8.603	0.017±0.025	0.079±0.104	0.444±0.541	0.668±1.416	0.019±0.017###***	0.135±0.133
14	Enterorhabdus	genus	0.535±0.390	0.934±1.350	0.819±0.492	1.452±0.773	1.080±2.045	0.991±0.940	1.171±1.010	1.695±1.917	1.215±0.427	0.639±0.634
15	norank_f__Bacteroidales_S24-7_group	genus	2.776±4.354	0.225±0.308	0.643±1.235	1.289±1.743*	0.171±0.220	0.157±0.220	0.884±1.259	1.594±1.700	1.030±1.455	0.765±0.739
16	unclassified_f__Erysipelotrichaceae	genus	0.008±0.005	0.369±0.470	0.001±0.001	0.483±0.628	1.107±2.283	0.041±0.057	0.322±0.391	0.112±0.161	6.510±11.100	0.003±0.002
17	Streptococcus	genus	1.449±1.118	0.324±0.187	0.951±1.287	1.172±0.678#	0.416±0.492	0.441±0.417	0.365±0.338	0.966±1.389#	0.364±0.561#	0.872±0.560
18	Enterococcus	genus	0.843±1.235	0.010±0.010	0.012±0.027	0.018±0.021	0.006±0.009	0.007±0.012	0.002±0.003	0.015±0.016	0.025±0.020	6.365±8.802
19	unclassified_k__norank	genus	0.352±0.109	0.239±0.321	0.186±0.277	1.452±2.541	0.570±0.550	0.476±1.029	0.207±0.168	1.225±1.017	0.257±0.256	0.480±0.686
20	Desulfovibrio	genus	0.205±0.256	0.133±0.175	0.642±1.058	0.743±0.901#*	0.037±0.034	0.288±0.423	0.735±0.860	0.596±0.658	1.055±1.429	0.403±0.540
21	Lactococcus	genus	0.080±0.098	0.088±0.094	0.048±0.044	0.949±0.879	0.067±0.139	0.109±0.083	0.406±0.399	0.509±0.294	0.225±0.127	1.934±3.340
22	Pseudomonas	genus	0.001±0.002	0.015±0.029	0.008±0.004	0.029±0.049	0.064±0.119	0.286±0.630	0.083±0.130	0.151±0.188#*	0.023±0.050	2.997±6.693
23	Lachnospiraceae_NK4A136_group	genus	0.086±0.095	0.086±0.113	1.072±2.064	0.286±0.332	0.043±0.056	0.402±0.463	0.104±0.075	0.129±0.069	0.299±0.541	0.087±0.115
24	Staphylococcus	genus	0.200±0.320	0.043±0.073	0.008±0.008	1.424±2.440	0.034±0.050	0.008±0.003	0.033±0.029	0.064±0.074	0.008±0.007	0.647±0.823
25	norank_c__Cyanobacteria	genus	0.003±0.004	0.046±0.090	0.013±0.015	1.842±4.091	0.045±0.090	0.076±0.161	0.065±0.099	0.045±0.071	0.012±0.024	0.011±0.020
26	F/B (firmicutes/Bacteroidetes)		21.813	278.143	99.293	46.265	298.870	337.026	60.246	35.768	70.338	59.341

LP (loperamide), H (high dose of FengLiao), M (medium dose of FengLiao), L (low dose of FengLiao). 1 g/ml FengLiao (H), 0.5 g/ml FengLiao (M), 0.25g/ml FengLiao (L) and 5mg/kg loperamide were pretreated in CastorOil_H, H, CastorOil_M, M, CastorOil_L, L, CastorOil_LP, LP groups once a day for three days respectively. 30 min after the last drug administration, 0.5ml castor oil was used to induce diarrhea in CastorOil_H, CastorOil_M, CastorOil_L and CastorOil_LP groups for 4 h and the intestinal contents were sequenced for bacterial 16S rRNA. n = 5, `x±SD. Vs model group *p<0.05, ** p<0.01, ***p<0.01, vs control group #p<0.05, ##p<0.01, ###p<0.001.

## Discussion

In this study, significant changes in the expression levels of AQP3, NHE3, NHE8, CRP, AGP and TRF were determined in mice with castor oil-induced diarrhea. FengLiao mitigated castor oil-induced diarrhea, most probably by down-regulating the expression levels of AQP3, NHE8 and the APP genes, including AGPs and CRP, and up-regulating TRF expression. In contrast, NHE2, NHE3, AQP4 and ALB expression levels played a limited role in this diarrhea model.

Studies have shown that AQP3 levels were down-regulated in mice with enterotoxigenic *Escherichia coli* (ETEC)-induced diarrhea [36], and up-regulated in diarrheal models induced by LPS [18], MgSO4 [37] and *Euphorbia pekinensis* [38, 39]. It has been reported that high levels of AQP3 results in the release of water from the lumen into the colonic interstitium [38], while inflammation and over-expression of AQP can synergistically disrupt the electrolyte balance of the intestine [39]. Furthermore, NHE3 and NHE8 have been found to be up-regulated in the jejunum of LPS-induced diarrheal mice [40], while NHE8 was found to be down-regulated in enteropathogenic *Escherichia coli* (EPEC)-induced diarrheal mice [41]. Thus, the expression of these proteins depends on the etiology of diarrhea, but all three of these genes were up-regulated in castor-induced diarrheal mice. CRP and AGP are positive acute phase proteins and their expressions increase rapidly during trauma and infection, while the expression of the negative acute phase protein, TRF, decreases [42]. Our results were found to be concurrent with these findings.

Castor oil significantly altered the gut microbiota. FengLiao increased the relative abundance of Bacteroidetes without affecting that of Firmicutes. Both Bacteroidetes and Firmicutes are major intestinal phyla that regulate inflammation and the immune status of the host [43]. Firmicutes plays an important role in host metabolism [44], but its excessive growth in the intestine increases the production of metabolic endotoxins. These metabolic edotoxins, such as lipopolysaccharides, can enter the bloodstream and trigger systemic inflammation [45]. On the other hand, Bacteroidetes decreased intestinal and systemic inflammatory responses [46, 47]. Therefore, an elevated F/B ratio is indicative of an inflammatory environment and immunological imbalance, which is characteristic of autoimmune disorders [46, 47]. Castor oil increased F/B, while both loperamide and lower doses of FengLiao exerted an ameliorative effect. Thus, the maintenance of a low F/B ratio may be involved in the anti-diarrheal mechanism of FengLiao. Compared with the anti-diarrheal effect of loperamide, FengLiao significantly increased the relative abundance of *Aerococcus*, *Corynebacterium_1*, *Pseudomonas* and *Bifidobacterium*, which are known to provide protection against infections [48]. Thus, FengLiao could also help to restore the health of the intestinal.

In conclusion, FengLiao provided protection against castor oil-induced diarrhea by altering the gut microbiota, inflammatory factors and intestinal transport proteins.

## Conclusion

FengLiao ameliorated castor oil-induced diarrheal symptoms and restored the dysregulation of AQP3, NHE3, NHE8, CRP, AGP and TRF expressions. Furthermore, FengLiao increased the abundance of *Bifidobacterium*, *Aerococcus*, *Corynebacterium_1* and *Pseudomonas*, and maintained a low F/B ratio to safeguard the balance between intestinal immunity and function.

## Supporting information

S1 File (RAR)
