# Correction: FengLiao affects gut microbiota and the expression levels of Na+/H+ exchangers, aquaporins and acute phase proteins in mice with castor oil-induced diarrhea

**DOI:** 10.1371/journal.pone.0243470

**Published:** 2020-12-03

**Authors:** Wenlu Chen, Xinyu Peng, Jingxian Yu, Xuanxuan Cheng, Minggui Yuan, Rong Xiang, Limei He, Danni Yu, Huahua Kang, Yufang Pan, Zhihong Xu

The fourth author's name is spelled incorrectly. The correct name is: Xuanxuan Cheng. The correct citation is: Chen W, Peng X, Yu J, Cheng X, Yuan M, et al. (2020) FengLiao affects gut microbiota and the expression levels of Na+/H+ exchangers, aquaporins and acute phase proteins in mice with castor oil-induced diarrhea. PLOS ONE 15(7): e0236511. https://doi.org/10.1371/journal.pone.0236511
